# Effect of Temperature on the Tear Fracture and Fatigue Life of Carbon-Black-Filled Rubber

**DOI:** 10.3390/polym11050768

**Published:** 2019-05-01

**Authors:** Wenbo Luo, Ming Li, Youjian Huang, Boyuan Yin, Xiaoling Hu

**Affiliations:** 1 Hunan Key Laboratory of Geomechanics and Engineering Safety, Xiangtan University, Xiangtan 411105, China; 2 College of Civil Engineering and Mechanics, Xiangtan University, Xiangtan 411105, China; lm_xtu@163.com (M.L.); byhyj@21cn.com (Y.H.); yinboyuanxtu@163.com (B.Y.)

**Keywords:** fatigue life, CB filled rubber, tear energy, crack growth rate, temperature dependence

## Abstract

The mechanical behaviour of carbon-black (CB)-filled rubber is temperature-dependent. It is assumed that temperature affects the fatigue life of rubber products by changing the tear energy of the material. The static tearing behaviour and fatigue crack propagation behavior of CB-filled rubber at different temperatures were investigated in this study. The critical tear energy of the material was measured through static tear fracture tests at different temperatures; it is shown that the critical tear energy decreases exponentially with increasing temperature. A fatigue crack growth test of a constrained precracked planar tension specimen was conducted at room temperature; the measurements verify that the fatigue crack growth follows a Paris–Erdogan power law. Considering the temperature dependence of the critical tear energy, the temperature dependent fatigue crack growth kinetics of CB-filled rubber was established, and the fatigue life of the material at high temperatures was predicted based on the kinetics. The predictions are in good agreement with experimental measurements.

## 1. Introduction

The excellent mechanical properties of rubber enable its widespread use in many applications [1,2,3]. Many rubber components, such as tires, vibration isolators, and impact bumpers, experience cyclic loading, which could lead to fatigue failure. Hence, estimating the fatigue life of rubber components has become a highly important industrial issue. To prevent the fatigue failure of rubber components in service, studies focusing on the durability of rubber have been increasing significantly for the past few years [4,5,6,7,8,9], and the prediction of fatigue failure in rubber has become very important [7,8,9].

During fatigue loading, due to the self-heating of the rubber and environmental conditions, rubber components can reach temperatures up to 110 °C [10]. Several studies have investigated the effect of temperature on the fatigue behaviour of rubber materials. For example, Lake and Lindley showed that the fatigue life of styrene butadiene rubber (SBR) gum decreases by a factor of 10,000 when the temperature increases from 0 to 100 °C, and the fatigue life of natural rubber (NR) gum decreases by a factor of 4 for the same temperature range [11]. Wu showed that the fatigue life of NR decreases with increasing temperature, and it decreases obviously when the temperature is higher than 75 °C [12]. An increase in temperature leads to a decline in the anti-fatigue property of the rubber. Therefore, the effective prediction of the fatigue life at different temperatures is of great significance for the timely replacement and extension of the service life of rubber products.

Although fatigue life prediction at different temperatures is very important for rubber components to ensure their reliability and safety, more research is needed on the fatigue life prediction considering temperature factors. To better understand and predict the fatigue life of rubber components, the temperature dependence of the critical tear energy is experimentally determined in the present study; the fatigue crack growth kinetics and fatigue life prediction model at high temperatures are also developed based on the fatigue crack growth law at room temperature.

## 2. Materials and Testing

### 2.1. Basic Mechanical Behaviour 

**Figure 1:**
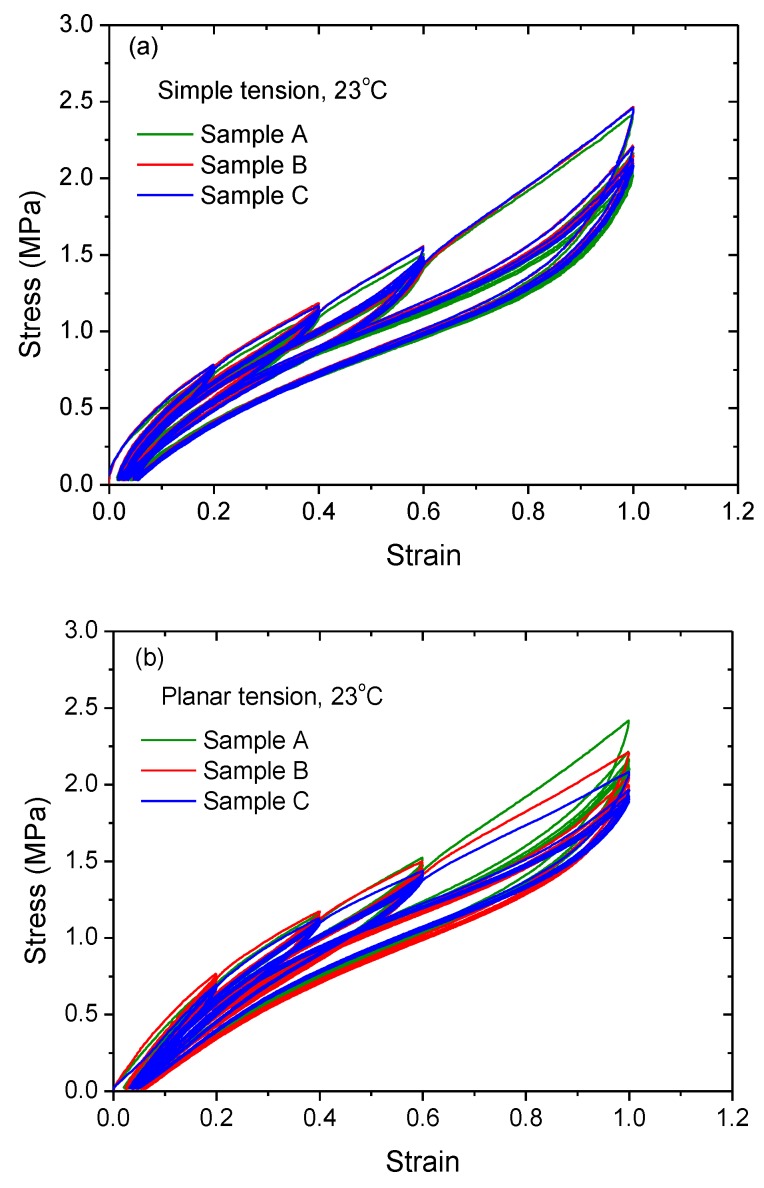

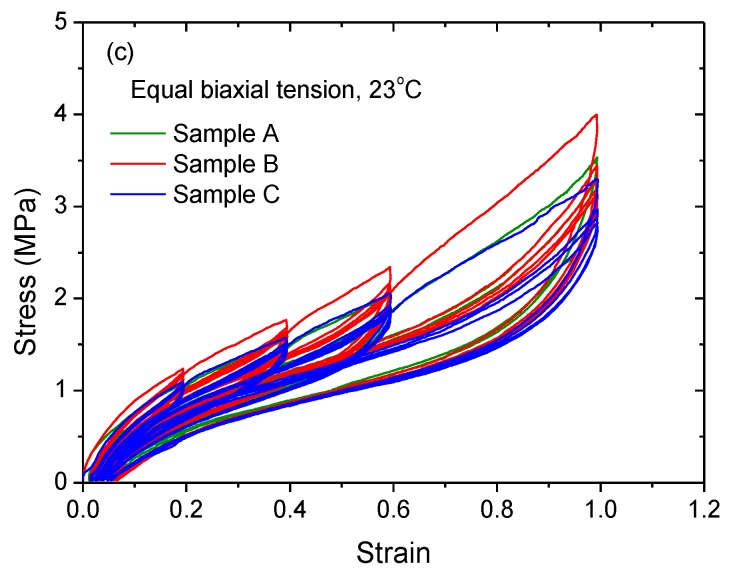
Loading/unloading stress-strain curves of carbon-black (CB)-filled rubber under (**a**) simple tension, (**b**) planar tension and (**c**) equal biaxial tension at 23 °C.

The tested material was CB-filled natural rubber with a shore-A hardness of 60, generously provided by the Zhuzhou Times New Material Technology Co., Ltd. in China. The main formulation of the rubber compound was as follows: 100 phr NR (Thailand RSS3), 42 phr carbon black (N330), 5 phr zinc oxide, 2 phr antioxidant, 2.2 phr sulfur, 2 phr stearic acid and 0.8 phr vulcanization activator. To understand the basic mechanical properties of the material, simple tension, planar tension and equal biaxial tension tests were carried out. The stress–strain curves for these tests are shown in Figure 1a–c, respectively.

### 2.2. Tear Fracture Tests

**Figure 2:**
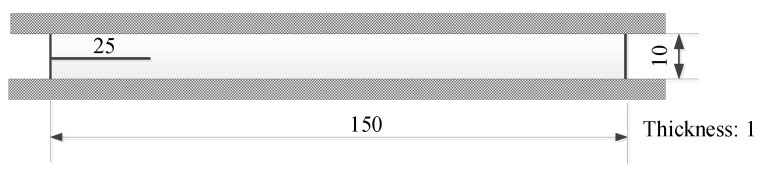
Dimensions of the tearing test specimen.

A high temperature shortens the fatigue life of rubber products. The main reason for this is that an increasing temperature reduces the crack propagation resistance of the material. According to Thomas [13], the tear energy of a rubber material is defined as the energy spent per unit thickness per unit increase in crack length:

(1) $$T_{a}=-{\left(\frac{\partial W}{\partial A}\right)}_{u}$$

where $T_{a}$ is the tear energy (or energy release rate), *W* is the elastic energy stored in the specimen, and A is the area of one surface of the crack. The suffix *u* denotes differentiation with constant displacement of the boundaries on which forces are applied. For a constrained precracked specimen, which consists of a wide strip of rubber material whose long side edges are attached to rigid grips, as shown in Figure 2, the tear energy is given by

(2) $$T_{a}=wh_{0}$$

where $h_{0}$ is the specimen height and w is the storage energy density of the specimen under constrained tension, which can be found from the stress–strain curve of the specimen.

The critical tear energy $T_{c}$ is a criterion index to determine if the crack is unstable. To obtain the temperature dependence of $T_{c}$ for CB-filled rubber, the rubber specimens were stretched at a strain rate of 0.01 s^−1^ at four different temperatures (−40, 23, 40 and 70 °C) to complete fracture on an Instron tensile testing machine. The tear fracture tests at each specified temperature were repeated three times.

### 2.3. Fatigue Crack Growth Tests (23 °C)

Fatigue crack growth tests were performed on an MTS 810 machine at an ambient temperature of 23 °C by applying fatigue loads in displacement-controlled mode to the specimens, as shown in Figure 2, to obtain the relationship between the crack growth rates and tear energy. The specimens were stretched by a prescribed sinusoidal pulse with a maximum strain $\varepsilon_{\max}$ and a minimum strain $\varepsilon_{\min}$ at a frequency of 3 Hz, and the crack growth rates were obtained by measuring the changes in the crack contour length with fatigue cycles. 

**Figure 3:**
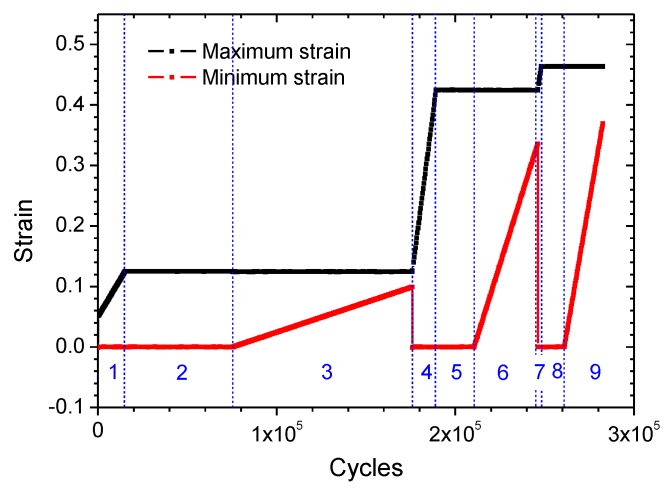
Graphical strain loading history.

The strain loading history, which comprises 9 stages, is shown in Figure 3. The 9 stages can be categorized into 3 loading types: (i) when strain ratio *R* = 0 with increasing $\varepsilon_{\max}$, such as in stages 1, 4, 7; (ii) when *R* = 0 while $\varepsilon_{\max}$ remains constant, such as in stages 2, 5, 8; (iii) when 0<R≤0.8 and $\varepsilon_{\max}$ remains constant while $\varepsilon_{\min}$ increases gradually, such as in stages 3, 6, 9. The detailed strain history is listed in Table 1.

## 3. Test Results and Discussion

### 3.1. Temperature Dependence of Critical Tear Energy

**Figure 4:**
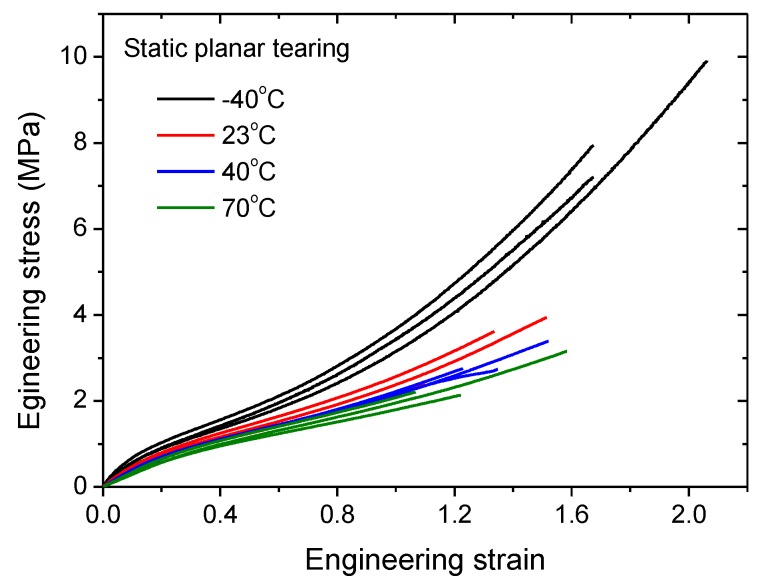
Planar tearing stress–strain curves at four different temperatures.

**Figure 5:**
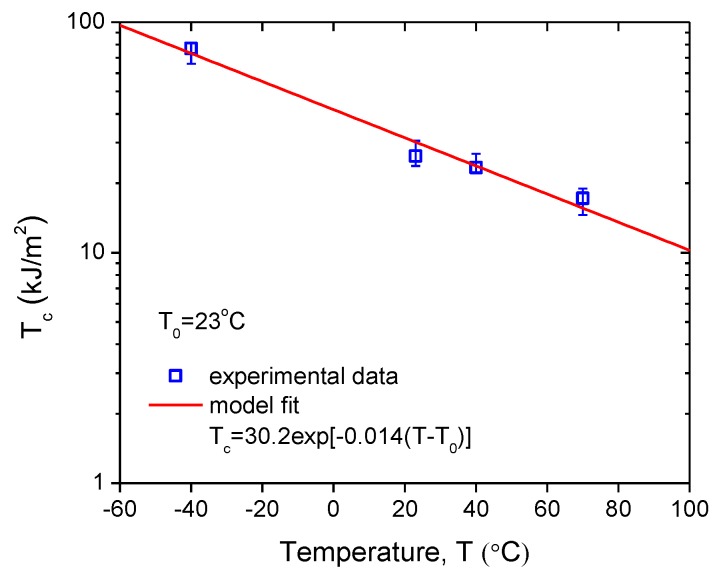
Variation of critical tear energy with temperature.

The stress–strain curves for the tear fracture tests at four specified temperatures are shown in Figure 4. The critical tear energy is calculated by Equation (2), and the averaged values for different temperatures are plotted in Figure 5. It is obvious that the critical tear energy decreases exponentially with increasing temperature. Thus, the critical tear energy at temperature *T* can be expressed as follows:

(3) $$T_{c}=T_{c,\mathrm{ref}}\exp\left[-k\left(T-T_{0}\right)\right]$$

where $T_{c,\mathrm{ref}}$ denotes the critical tear energy at a reference temperature $T_{0}$ and *k* measures the degree of the temperature sensitivity.

Fitting the experimental data in Figure 5 by Equation (3) with *T*_0_ = 23 °C yields *k* = 0.014/°C and *T*_c,ref_ = 30.2 kJ/m^2^. The correlation coefficient *r* = 0.994, which indicates that the model fit the experimental data well. Thus, the critical tear energy of the tested CB-filled rubber was related to the temperature, as described by Equation (4):

(4) $$T_{c}=30.2\exp\left[-0.014\left(T-T_{0}\right)\right]$$

### 3.2. Fatigue Crack Growth at 23 °C

**Figure 6:**
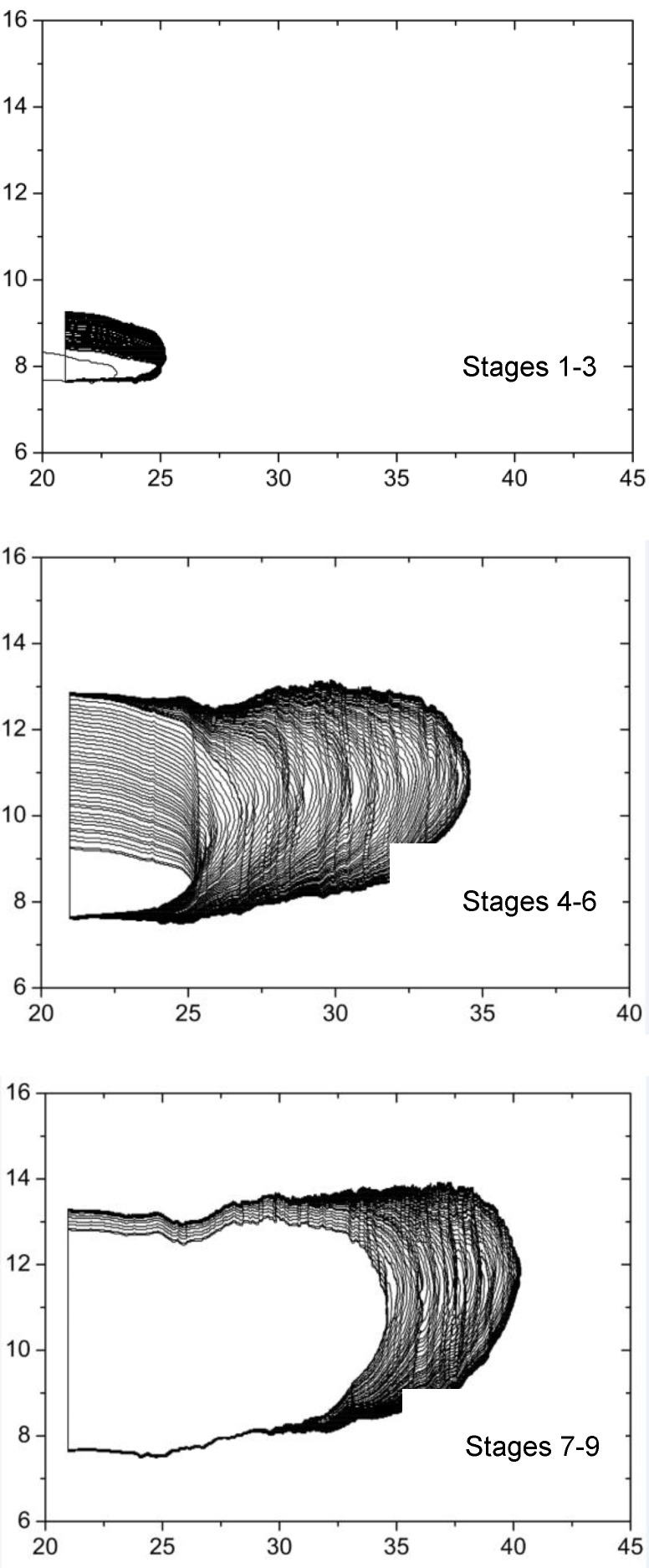
Crack front profiles for different load stages.

**Figure 7:**
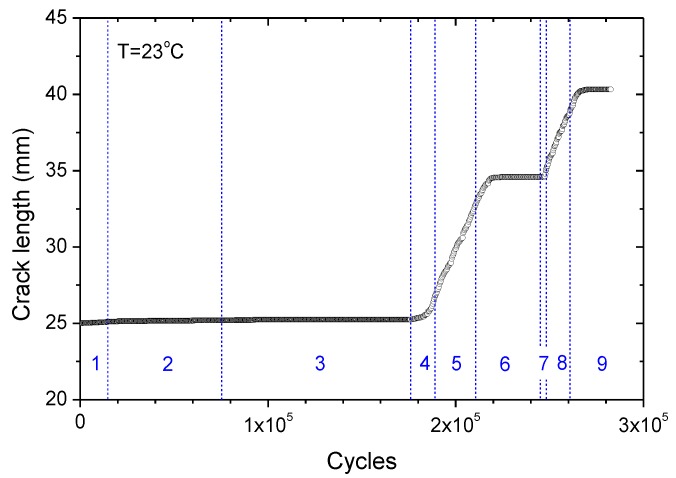
Crack contour length as a function of fatigue cycles for CB-filled rubber at 23 °C.

**Figure 8:**
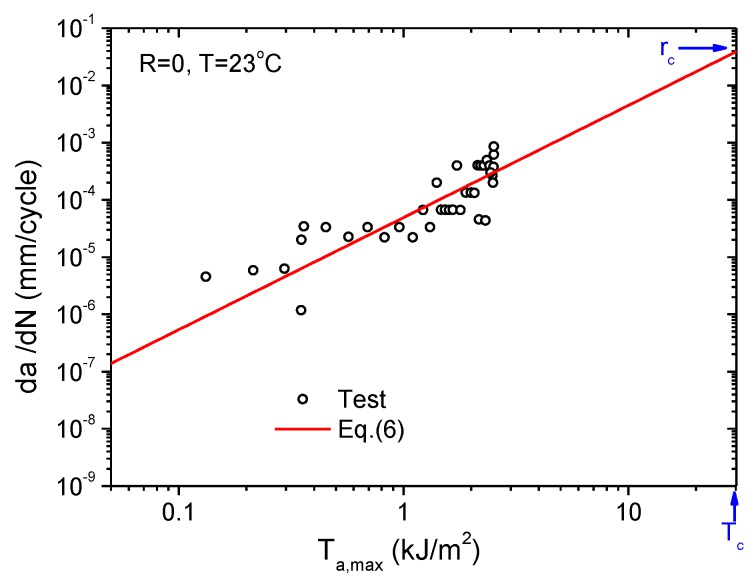
Relationship between crack growth rate and maximum tear energy.

The specimen used for fatigue crack growth testing is the same as those used in tear fracture tests, and it was subjected to cyclic strain as depicted in Figure 3. Figure 6 shows the corresponding crack front profiles for different loading stages, from which the crack length can be determined for any specified load cycle by image analysis, as shown in Figure 7. Consequently, the crack growth rate d*a*/d*N* can also be determined by differentiating the crack length with respect to load cycle, and plotted as a function of the corresponding tear energy $T_{a,\max}$, which is the driving force for crack growth corresponding to a maximum strain $\varepsilon_{\max}$ in a fatigue cycle. For an intermediate tear energy region, e.g., 0.3 kJ/m^2^ ≤ *T*_a,max_ < 10 kJ/m^2^, the relation of $da/dN∼T_{a,\max}$ for rubbers often approximates the Paris–Erdogan power law from [14]:

(5) $$\frac{da}{dN}=r_{c}\cdot {\left(\frac{T_{a,\max}}{T_{c}}\right)}^{F}$$

where $r_{c}$ denotes the critical crack growth rate. The determination of the model parameters requires curve fitting of the double logarithmic correlation of the crack growth rate and the tear energy. Figure 8 shows the measured $da/dN∼T_{a,\max}$ plot of the tested rubber on a double logarithmic scale. Recalling *T*_c_ = 30.2 kJ/m^2^, as obtained in the static tear fracture test at 23 °C, the Paris–Erdogan parameters, $r_{c}$ and *F*, for the tested CB-filled rubber can be determined by fitting the plotted points in Figure 8 with Equation (5). The resultant fatigue crack growth kinetics for *R* = 0 at 23 °C is then expressed by Equation (6), where *r*_c_ = 0.039 mm/cycle, *F* = 1.96.

(6) $$\frac{da}{dN}=0.039{\left(\frac{T_{a,\max}}{30.2}\right)}^{1.96}=4.90\times 10^{-5}{\left(T_{a,\max}\right)}^{1.96}$$

## 4. High-Temperature Fatigue Life Prediction

**Figure 9:**
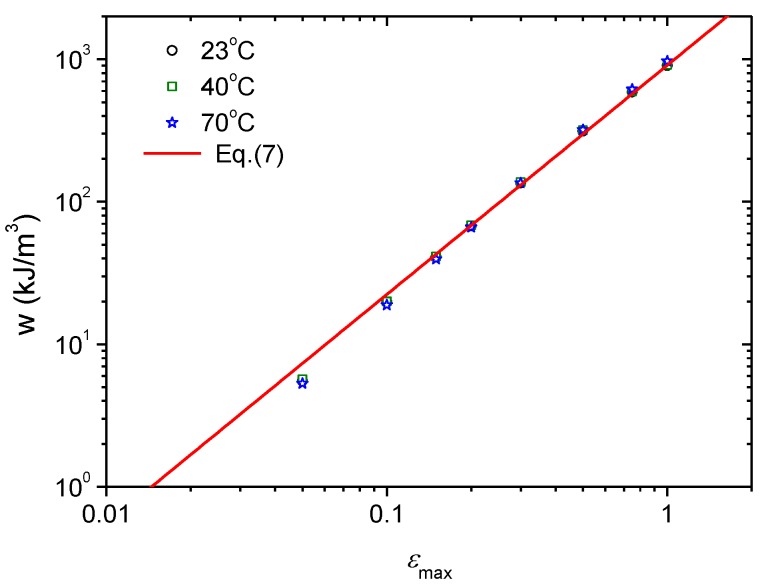
Variation of the storage energy density with maximum strain at different temperatures.

Equation (5) shows that the fatigue crack growth rate depends on both the tear energy ($T_{a,\max}$), which is the crack driving force, and the critical tear energy ($T_{c}$), which is the crack growth resistance. In the previous section, the temperature dependence of $T_{c}$ was obtained, as given by Equation (4). Because of the viscoelasticity of CB-filled rubber, it is generally accepted that the storage energy density of the material under cyclic loading is also temperature-dependent. That is, the tear energy ($T_{a,\max}$) should vary with temperature. For the cyclic planar tension of the constrained precracked specimen, the tear energy is the energy released during unloading. It is expressed by Equation (2), and its value is independent of crack length; therefore, the storage energy density in cyclic deformation is also independent of crack length but varies with temperature and the experienced maximum strain. Figure 9 shows the experimental data for the variation of the storage energy density of the CB-filled rubber specimen that experienced a maximum strain at 23, 40 and 70 °C. The temperature dependence of the storage energy density is very weak because the glass transition temperature, *T*_g_, of the CB-filled rubber material is below −41 °C, as measured from the temperature sweep tests of dynamic mechanical analysis at various load frequencies ranging from 1 to 50 Hz by Hu et al. [15], while the test temperatures in Figure 9 are far higher than the *T*_g_. In the test temperature range, CB-filled rubber is hyperelastic rather than viscoelastic in nature, so the temperature dependence of the storage energy density is also very weak and negligible in predicting the high-temperature fatigue life of the CB-filled rubber.

Figure 9 shows that the storage energy density for test temperatures above 23 °C is linearly related to the maximum strain on a double logarithmic scale; thus, the power law is as follows:

(7) $$w\left(\varepsilon_{\max}\right)=908.15\times \varepsilon_{\max}^{1.608}$$

The tear energy for fatigue loading is thus obtained by substituting Equation (7) into Equation (2):

(8) $$T_{a,\max}\left(\varepsilon_{\max}^{}\right)=w\left(\varepsilon_{\max}^{}\right)\cdot h_{0}=908.15\times 0.01\times \varepsilon_{\max}^{1.608}=9.08\varepsilon_{\max}^{1.608}$$

The temperature-dependent fatigue crack growth of the constrained CB-filled rubber specimen at high temperatures beyond 23 °C can then be quantitatively estimated by combining Equations (4), (8) and (5):

(9) $$\begin{array}{cc} \frac{da\left(\varepsilon_{\max},T\right)}{dN} & =r_{c}\cdot {\left[\frac{T_{a,\max}\left(\varepsilon_{\max}\right)}{T_{c}\left(T\right)}\right]}^{F}=r_{c}\cdot {\left[\frac{T_{a,\max}\left(\varepsilon_{\max}\right)}{T_{c,\mathrm{ref}}\cdot e^{-k\left(T-T_{0}\right)}}\right]}^{F}=r_{c}\cdot {\left[\frac{T_{a,\max}\left(\varepsilon_{\max}\right)}{T_{c,\mathrm{ref}}}\right]}^{F}\cdot e^{F\cdot k\cdot \left(T-T_{0}\right)}\\ & =0.039\cdot {\left[\frac{9.08\varepsilon_{\max}^{1.608}}{30.2}\right]}^{1.96}\cdot e^{1.96\times 0.014\cdot \left(T-T_{0}\right)}=0.0037\cdot \varepsilon_{\max}^{3.152}\cdot e^{0.0274\left(T-T_{0}\right)} \end{array}$$

**Figure 10:**
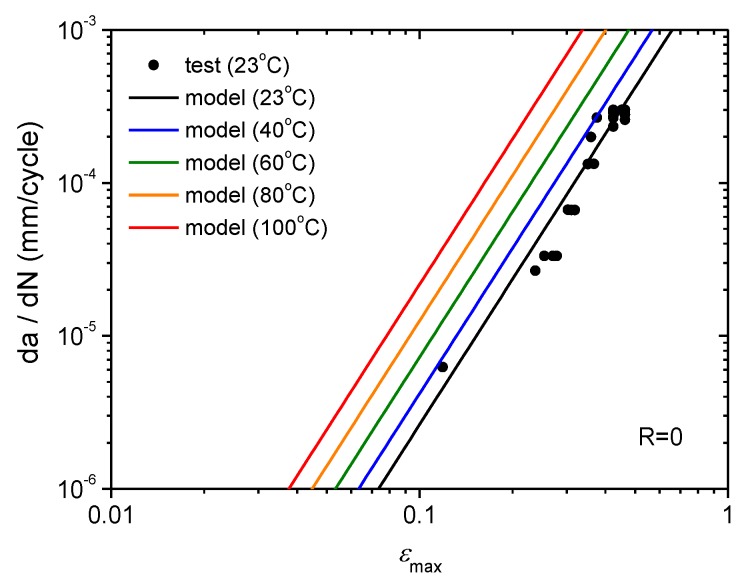
Predictions of fatigue crack growth rate at temperatures above 23 °C.

The fatigue crack growth rate predictions by Equation (9) for 23, 40, 60, 80 and 100 °C are shown in Figure 10 by solid lines with different colours. The test data for 23 °C are also plotted in the same figure to verify the model.

For the cyclic simple tension of a single edge notched specimen, the tear energy depends on the gauge section strain energy density *w*, the crack length, *a*, and a deformation dependent parameter *q* [16]:

(10) $$T_{a,\max}=2q\cdot w\left(\varepsilon_{\max}\right)\cdot a$$

Substituting Equation (10) into Equation (5) and recalling the temperature dependency $T_{c}\left(T\right)$, the fatigue crack growth rate at different temperatures can be expressed as follows:

(11) $$\frac{da\left(\varepsilon_{\max},T\right)}{dN}=r_{c}\cdot {\left[\frac{T_{a,\max}\left(\varepsilon_{\max}\right)}{T_{c}\left(T\right)}\right]}^{F}=B{\left[2q\cdot w\left(\varepsilon_{\max}\right)\right]}^{F}a^{F}\cdot e^{F\cdot k\cdot \left(T-T_{0}\right)}$$

where $B=r_{c}\cdot T_{c,\mathrm{ref}}^{-F}$.

The fatigue life can be obtained by integrating Equation (11) as follows:

(12) $$N_{f}=\frac{1}{F-1}\frac{1}{B{\left[2q\cdot w\left(\varepsilon_{\max}\right)\right]}^{F}}e^{-F\cdot k\cdot \left(T-T_{0}\right)}\left[\frac{1}{a_{0}^{F-1}}-\frac{1}{a_{f}^{F-1}}\right]$$

where $a_{0}$ is the initial crack length, which usually defines the intrinsic flaw size in the material or the typical size of the nucleated fatigue crack, and was set to 0.006 mm in this study; $a_{f}$ is the critical final crack length corresponding to the final fatigue failure. In the case that $a_{f}\gg a_{0}$, the fatigue life becomes independent of the critical crack length.

(13) $$N_{f}=\frac{1}{F-1}\frac{1}{B{\left[2q\cdot w\left(\varepsilon_{\max}\right)\right]}^{F}}e^{-F\cdot k\cdot \left(T-T_{0}\right)}\frac{1}{a_{0}^{F-1}}$$

**Figure 11:**
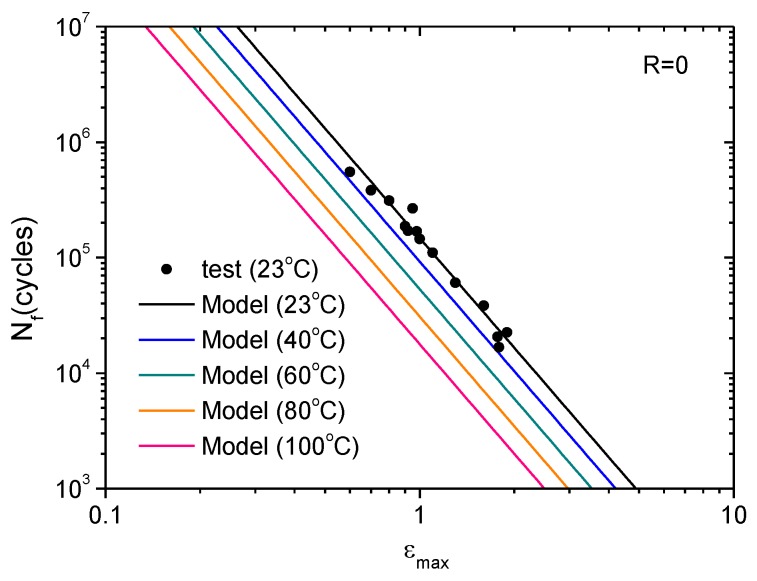
Variation of fatigue life at different temperatures with maximum strain: predictions vs. tests.

**Figure 12:**
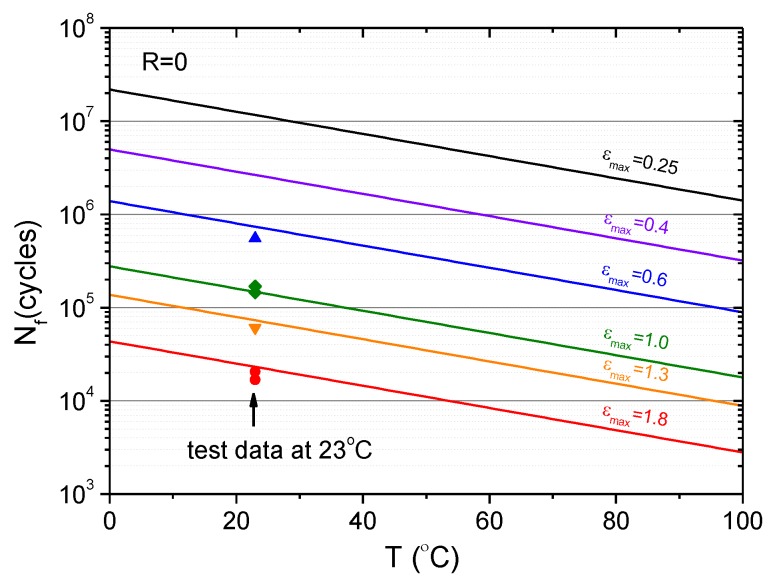
Variation of fatigue life under different maximum strains with temperature: predictions vs. tests.

Recalling the model parameters for the investigated CB-filled rubber material, namely *F* = 1.96, *r*_c_ = 0.039 mm/cycle, *T*_c,ref_ = 30.2 kJ/m^2^, and *k* = 0.014/°C, and setting *q* = 2.5 for the intermediate strain range, the fatigue cycles that cause a specified final crack length can be calculated by Equation (12) for different temperatures. The calculated results are shown in Figure 11 for a final crack length of 1 mm, which is the critical flaw size for a fatigue safety inspection of rubber components based on a visual inspection. The fatigue life predicted by Equation (12) is in good agreement with the test data at 23 °C. Figure 12 shows the model prediction of the variation of fatigue life under different maximum strains and temperatures.

## 5. Conclusions

For an intermediate strain range and high temperatures in which only the temperature dependence of the critical tear energy is of concern, the fatigue crack growth rate of CB-filled rubber follows the Paris–Erdogan power law with respect to the maximum strain, and the critical tear energy decreases exponentially with increasing temperature. Consequently, the fatigue life decreases with increases in temperature and strain. 

Based on the viewpoint that temperature affects the fatigue life of rubber products by changing the tear energy of the material, a model based on the tear fracture test data at different temperatures and fatigue crack growth test data at room temperature can be used to estimate the temperature-dependent fatigue life of the rubber studied in this paper. According to the proposed method, the fatigue life prediction of the CB filled rubber at different temperatures can be obtained without carrying out the corresponding fatigue tests. It can decrease the test time and reduce the research and development costs.

## Figures and Tables

**Table 1:** Detailed fatigue strain history.

Loading Stages	*R*	*ε*_min_	*ε*_max_	*N*/Cycles
1	0	0	0.05~0.125	0~15,000
2	0	0	0.125	15,000~76,000
3	0~0.8	0~0.10	0.125	76,000~176,000
4	0	0	0.125~0.425	176,000~190,000
5	0	0	0.425	190,000~210,000
6	0~0.8	0~0.34	0.425	210,000~246,000
7	0	0	0.425~0.4625	246,000~248,000
8	0	0	0.4625	248,000~261,000
9	0~0.8	0~0.37	0.4625	261,000~282,500
